# Alkylglycerol Derivatives, a New Class of Skin Penetration Modulators

**DOI:** 10.3390/molecules22010185

**Published:** 2017-01-22

**Authors:** Sergio Alberto Bernal-Chávez, Lilia Yazmín Pérez-Carreto, María Guadalupe Nava-Arzaluz, Adriana Ganem-Rondero

**Affiliations:** Division de Estudios de Posgrado (Tecnología Farmacéutica), Facultad de Estudios Superiores Cuautitlán, Universidad Nacional Autónoma de Mexico, Cuautitlán Izcalli 54740, Estado de Mexico, Mexico; q901108@hotmail.com (S.A.B.-C.); black_greenblue@hotmail.com (L.Y.P.-C.); lupita_navaarzaluz@yahoo.com.mx (M.G.N.-A.)

**Keywords:** alkylglycerols, skin permeation, absorption modulators, absorption retarders, transepidermal water loss (TEWL), attenuated total reflectance-Fourier transform infrared (ATR-FTIR) spectroscopy

## Abstract

The absorption modulating activity of two alkylglycerol derivatives (batyl and chimyl alcohol) on skin barrier properties was evaluated. Biophysical tests such as transepidermal water loss (TEWL) and attenuated total reflectance–Fourier transform infrared (ATR-FTIR) spectroscopy, as well as in vitro skin permeation studies, were performed in order to determine the effect of these compounds as chemical absorption modulators. Four drugs were used as models: three NSAIDS (diclofenac, naproxen, and piroxicam) and glycyrrhizic acid. The results showed that treatment of the skin with alkylglycerols caused (i) a reduction on the amount of drug permeated; (ii) a reduction in TEWL; and (iii) changes in the ATR-FTIR peaks of stratum corneum lipids, indicative of a more ordered structure. All of these findings confirm that alkyl glycerols have an absorption retarding effect on the drugs tested. Such effects are expected to give rise to important applications in the pharmaceutical and cosmetic sectors, in cases where it is desirable for the drug to remain in the superficial layers of the skin to achieve a local effect.

## 1. Introduction

It is fully recognized that one of the major drawbacks when administering drugs through the skin, especially when seeking a systemic effect, is the limited permeation of most of these drugs due to the presence of the barrier imposed by the structural characteristics of the stratum corneum (SC) [1]. In recent decades, different approaches have been tested in order to overcome the skin’s barrier, including mechanical and electrical disruption, as well as chemical modification (e.g., [2,3]). However, in specific cases, such as those with pesticides, chemical warfare agents, sunscreens, mosquito repellants, and SC targeted agents, where it is desirable for them to reside on the skin surface, an increase in SC impermeability is sought. While some penetration enhancers act on skin lipids, creating a more fluid environment and then promoting therapeutic agents to permeate, there are molecules with an opposite effect [4]. These molecules known as penetration retarders or reducers, may be useful by restricting the location of the drugs to the uppermost layers of the skin, not only in those cases where the target site is the surface of the skin, but also when absorption may lead to unwanted side effects or toxicity. Collectively, both enhancers and retarders are known as penetration modifiers [5]. Although the mechanisms of action of retarders have not been completely understood, it has been proposed that they may act (i) as antisolvents, by avoiding the partitioning of the drug into the SC; (ii) by adding lipid-like molecules into the lipid bilayers to stiffen the SC [6]; (iii) by providing order to the lipid–lipid arrangement in the SC and then decreasing the diffusion coefficient of the drug [5]. Two decades ago, Hadgraft et al. [4] conducted a study that examined the effect of Azone^®^ and five derivatives on the permeation of m-diethyltoluamide and metronidazole through the skin. They reported that one of the analogues, referred to as N-0915, behaved as a retarder. Stabilization of the lipid bilayers of dipalmitoylphosphatidylcholine multilamellar vesicles, with an increase in phase transition temperature, as well as the presence of H-bonding groups able to interact with adjacent ceramide head groups on either side, were proposed as mechanisms of action of N-0915.

**Figure 1:**
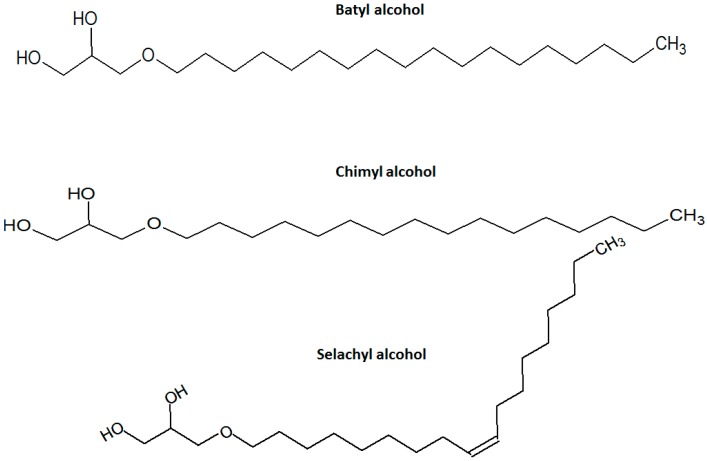
Chemical structures of alkylglycerol derivatives.

Alkylglycerols (glycerol alkyl ethers) are found in natural sources, mainly in shark liver oil. Major alkylglycerols (AGs), whose structures are shown in Figure 1, include chimyl (hexadecyl), batyl (octadecyl), and selachyl (*E*-octadec-9-enyl) ethers. Another source of 1-*O*-alkylglycerols may occur naturally as lipid ethers in cow’s milk, human milk, and blood-forming organs (bone marrow, liver, and spleen). AGs exhibit relevant therapeutic effects, such as antitumor activity, control of the immune response, as well as antibacterial and antifungal activity [7]. Furthermore, Fernandez et al. [8] demonstrated the enhancing ability of a mixture of 1-*O*-alkylglycerols and later, Bilbao et al. [9] showed, in an in silico study, that 1-*O*-alkylglycerols, particularly 1-*O*-octadecyl glycerol and 1-*O*-hexadecyl glycerol, may have important penetration enhancing activity, emphasizing the gastrointestinal absorption. In addition to these results, what especially caught our attention was the structure of these compounds (Figure 1), containing a long alkyl chain (16 and 18 carbons, saturated in the case of batyl and chimyl alcohol), and two hydroxyl groups, which may suggest an ability to be inserted between the intercellular lipids and to interact with their polar heads through H-bonding. Therefore, the objective of this work was to evaluate the penetration modulating activity of two AGs derivatives, batyl and chimyl, determining its effect on the cutaneous permeation barrier. Therefore, in vitro permeation studies using Franz type cells were performed in order to define the enhancer/retarder effect of these two compounds on the diffusion of four model drugs: diclofenac, piroxicam, naproxen, and glycyrrhizic acid ammonium salt. These drugs were selected because of their differences in some physicochemical properties such as molecular weight, log P, and water solubility (Table 1). Furthermore, attenuated total reflectance-Fourier transform infrared (ATR-FTIR) spectroscopy and transepidermal water loss (TEWL) helped to understand the effect of these modifiers on SC structure. The relevance of this work is that there are no reports on the use of these compounds as skin absorption modulators.

## 2. Results

### 2.1. In Vitro Skin Permeation Studies

**Figure 2:**
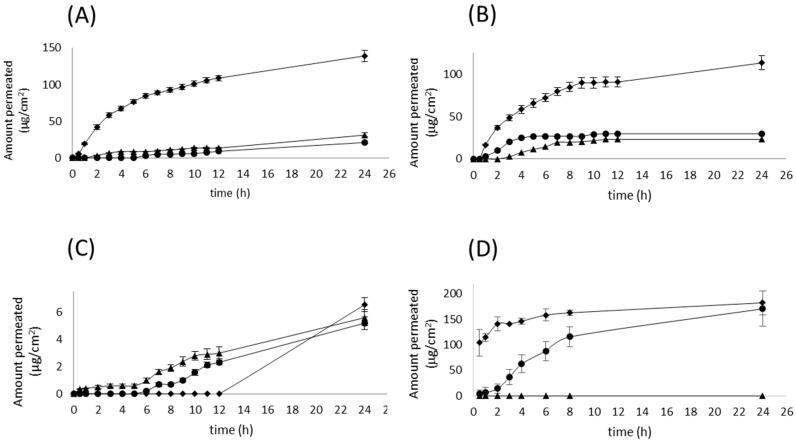
Effect of batyl and chimyl alcohol in Transcutol^®^ on drug permeation through excised pig skin. (**A**) Diclofenac; (**B**) Naproxen; (**C**) Piroxicam; (**D**) Glycyrrhizic acid ammonium salt. Transcutol^®^ (◆); batyl alcohol/Transcutol^®^ (●); chimyl alcohol/Transcutol^®^ (▲). Mean ± SD (*n* = 6).

**Figure 3:**
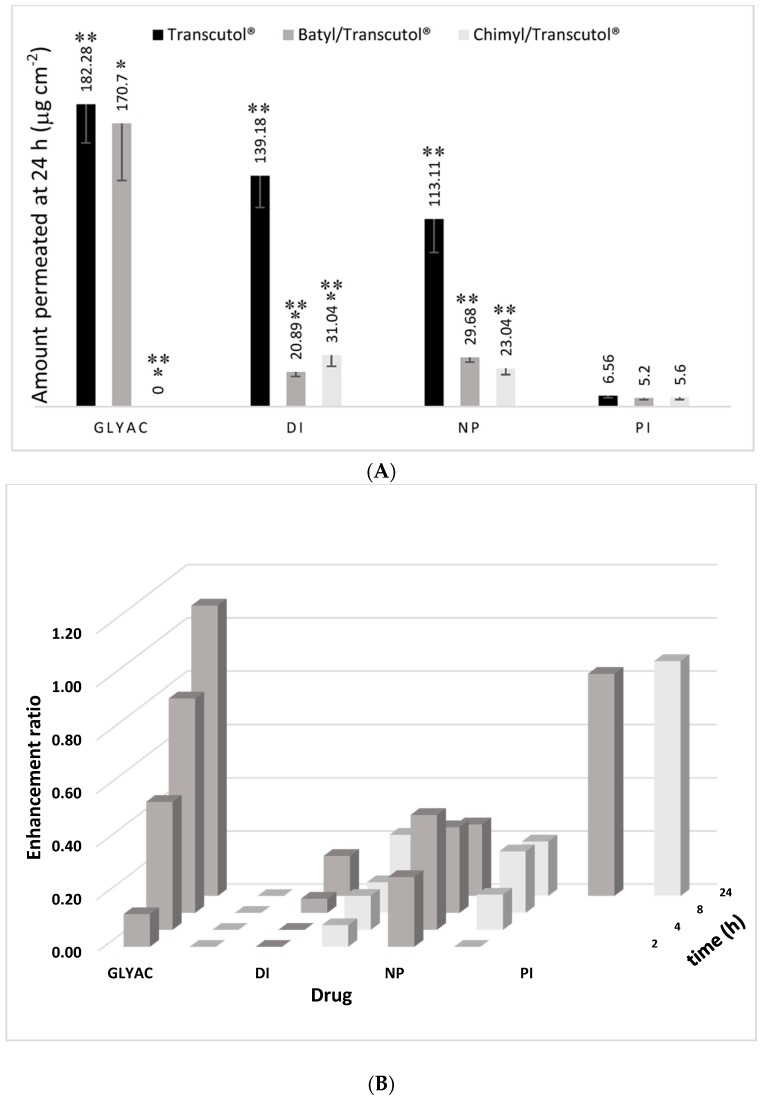
Comparison of the effect of batyl and chimyl alcohols: (**A**) On the total amount permeated (µg·cm^−2^) after 24 h. * *p* < 0.05 (batyl vs. chimyl); ** *p* < 0.05 (Transcutol^®^ vs. batyl or chimyl) by comparing each drug separately. (**B**) On the enhancement ratios at different times. Enhancement ratio = Amount permeated with alkylglycerol/Amount permeated without alkylglycerol. ■ Transcutol^®^; ■ Batyl alcohol/Transcutol^®^; ■ Chimyl alcohol/Transcutol^®^. GLYAC = glycyrrhizic acid; DI = diclofenac; NP = naproxen; PI = piroxicam. *p* < 0.05 for batyl vs. chimyl for all the times, with the exception of 24 h for DI, NP, and PI.

The permeation profiles of the four drugs with each AG derivative are shown in Figure 2. As shown, a reduction in the amount permeated of diclofenac, naproxen, and glycyrrhizic acid was observed when treating the skin with the two alcohols, batyl and chimyl. Another interesting feature is that similar profiles with flux values close to each other were obtained between the two AG derivatives for diclofenac, naproxen, and piroxicam. Only glycyrrhizic acid showed two very different profiles, finding that chimyl alcohol completely blocked the transport of this drug to the receptor solution. For a drug like piroxicam/Transcutol P^®^ able to reach the receptor medium only after 12 h, combination with batyl and chimyl alcohols enhanced the drug permeation notably after 6 h, where an increase in the flux was observed. However, at 24 h, the total amount of piroxicam permeated was lower (about 20% for batyl alcohol and 15% for chimyl alcohol) than with Transcutol P^®^ alone (Figure 3A).

The enhancement ratio (ER = Amount permeated with AG/Amount permeated without AG) for the four drugs was calculated at different times (Figure 3B). As shown, diclofenac, naproxen, and glycyrrhizic acid resulted in ER values less than one with the two AGs, even with zero values in some cases. Regarding piroxicam, ER was calculated only for the 24 h, because no drug was found in the receptor solution at shorter time intervals when piroxicam/Transcutol P^®^ was tested. It is important to mention the fact that both batyl and chimyl alcohols caused a reduction on the total amount permeated at 24 h for all the drugs tested (Figure 3A).

**Figure 4:**
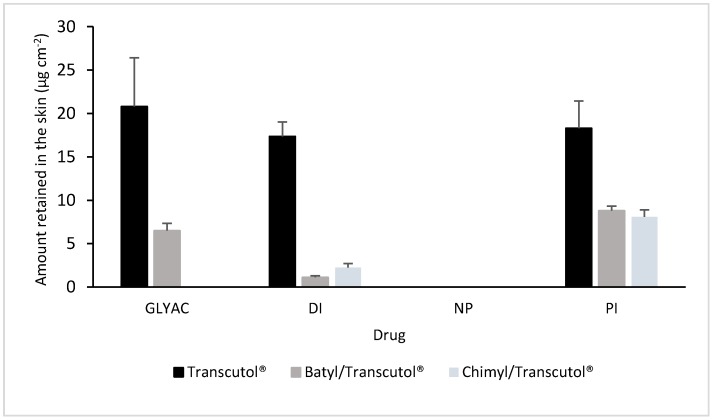
Amount of drug retained in the skin (µg·cm^−2^) after the permeation experiments using Transcutol^®^, batyl alcohol/Transcutol^®,^ and chimyl alcohol/Transcutol^®^ as solvents for the drugs. GLYAC = glycyrrhizic acid; DI = diclofenac; NP = naproxen; PI = piroxicam. *p* < 0.05 (Transcutol^®^ vs. batyl or chimyl) by comparing each drug separately; *p* < 0.05 for batyl vs. chimyl, with the exception of PI.

The results of the amount of drug retained in the skin are shown in Figure 4. The results are consistent with those found for the quantities permeated. Again, there is a reduction for the combinations AG/Transcutol^®^ compared with Transcutol^®^ alone. Indeed, in the case of naproxen, nothing was found in the skin (below the limit of quantification), even with Transcutol^®^. In the case of glycyrrhizic acid, there was nothing retained with chimyl alcohol, which is in agreement with the result of the amount permeated, where there was nothing in the receptor solution.

### 2.2. TEWL Measurement

**Figure 5:**
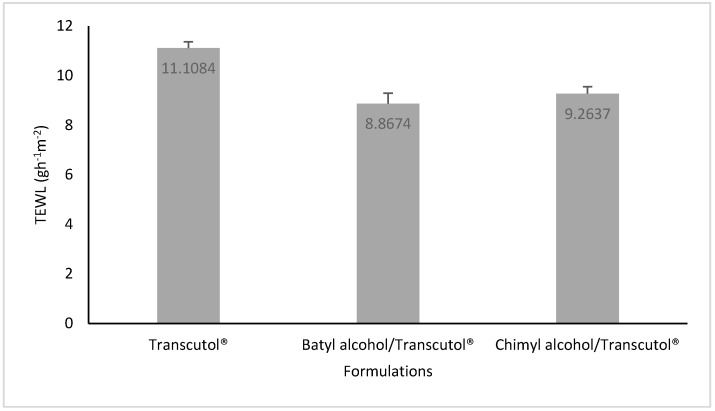
TEWL values for skin treated with Transcutol^®^, batyl alcohol/Transcutol^®^, and chimyl alcohol/Transcutol^®^. * *p* < 0.05 for Transcutol^®^ vs. batyl and chimyl alcohols.

Treatment of the skin with Transcutol P^®^ resulted in a TEWL increase of about 64.6%, compared with untreated skin (TEWL = 6.75 ± 0.03 gh^−1^·m^-2^ ). However, as shown in Figure 5, a decrease of about 20% and 16.6% was observed after the treatment with batyl and chimyl alcohol, respectively, compared with Transcutol P^®^.

### 2.3. ATR-FTIR Spectroscopy

**Figure 6:**
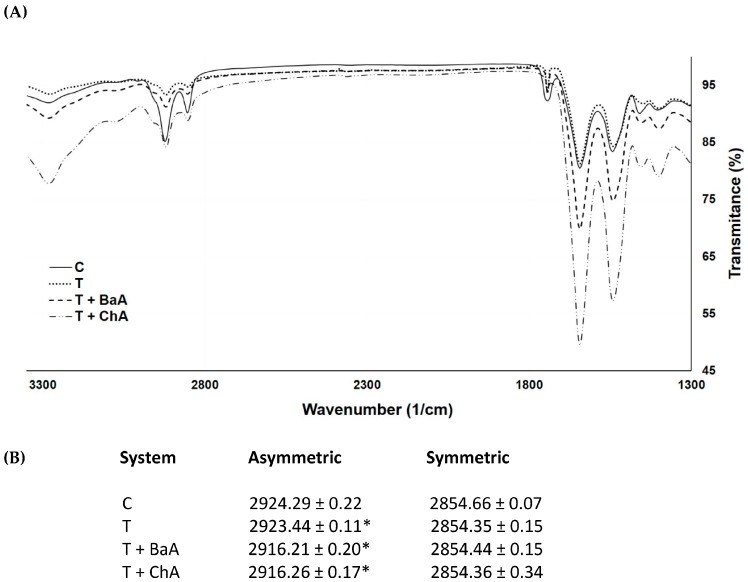
(**A**) Representative ATR-FTIR spectra of porcine ear skin. Untreated (control, C); treated with Transcutol^®^ (T); treated with batyl alcohol/Transcutol^®^ (T + BaA); treated with chimyl alcohol/Transcutol^®^ (T + ChA); (**B**) Frequencies of the CH_2_ asymmetric and symmetric stretching vibrations of the stratum corneum lipid acyl chains. Mean ± SD (*n* = 6). * *p* < 0.05.

The usefulness of ATR-FTIR spectroscopy in investigating the organization of SC lipids have been extensively demonstrated through the study of different vibrational frequencies, such as those occurring between 1475–1460 cm^−1^, related to the CH_2_ scissoring modes, as well as the symmetric stretching frequency, which ranges from 2847 to 2855 cm^−1^, and the asymmetric stretching band observed between 2915 and 2924 cm^−1^ [11,12]. The scissoring region contains peaks centered at appproximately 1473 and 1462 cm^−1^ (characteristic of orthorhombic phases), together with a peak at approximately 1468 cm^−1^ (characteristic of hexagonal phases). The spectral position of these vibrations provides information of the acyl chain packing of the lipids, as well as of conformational order, e.g., *trans-gauche* isomerization [13]. Thus, the effect of Transcutol P^®^, batyl alcohol/Transcutol P^®^, and chimyl alcohol/Transcutol P^®^ on the lipid alkyl chains was evaluated in vitro by the ATR-FTIR technique. In this work, we focused on the nearby peaks at 2850 cm^−1^ and 2920 cm^−1^ related to the symmetric and asymmetric stretching vibrations of the lipid alkyl chains, respectively. As can be seen in Figure 6, there was a shift to lower wavenumbers for the asymmetric stretching vibration for the three systems tested (Transcutol^®^, batyl alcohol/Transcutol^®^, and chimyl alcohol/Transcutol^®^) vs. the control (C). Statistical analysis (*t* test for paired samples) revealed a significant difference in the shift value for the asymmetric stretching mode (*p* < 0.05) in comparison to the control (untreated skin). No changes were observed in the case of the symmetric stretching vibration.

## 3. Discussion

In 1993, Fernandez et al. [8] studied the ability of a mixture of 1-*O*-alkylglycerols (chain lengths ranging 16–18 carbons) as absorption enhancers for the oral route, demonstrating an enhancing effect similar to that obtained with oleic and lauric acid. Later, Bilbao et al. [9] performed an in silico study corroborating the results of Fernandez et al. [8] through the determination of some physicochemical descriptors using the ModesLab**^®^** software. Despite being aware of the structural differences between the gastrointestinal epithelium and the skin, as well as of the mechanisms of transport involved, we had a strong curiosity about the effect of these compounds on skin permeability. Then, as an initial approach, four molecules (three analgesics:—diclofenac, naproxen, and piroxicam—and glycyrrhizic acid ammonium salt) were selected as models to test the enhancing ability of two AG derivatives: chimyl (hexadecyl) and batyl (octadecyl) alcohols. Transcutol P**^®^**, well known for its permeation enhancing ability (e.g., [14,15]), was chosen as the vehicle in order to solubilize both drugs and AGs. In vitro permeation studies, TEWL measurements and ATR-FTIR spectroscopy were used in an attempt to clarify the behavior of these compounds.

The results showed that, in general, for all drugs tested, both batyl and chimyl alcohols acted by reducing the total amount permeated at 24 h (Figure 3). Furthermore, the ER calculated at different times yielded values less than one. The exception was piroxicam, which showed an increase rather than a decrease in the transport for the first 6–12 h when combined with batyl and chimyl alcohols. Although the reasons for this are unclear, it may be related to the interactions between piroxicam and AGs or to the effect of the vehicle. Different studies have shown that enhancement and retarder effects depend notably on the vehicle used [5,16]. The results of the amount of drug extracted from the skin at the end of the permeation experiments confirmed that combination of AGs with Transcutol**^®^** reduced the amount of drug retained in the skin (Figure 4). Indeed, in the case of naproxen, the amount found with Transcutol**^®^** was below the limit of quantification; meanwhile, with glyciyrrhizic acid, the chimyl alcohol/Transcutol**^®^** combination completely blocked its entry into the skin, finding no drug in the receiver solution or in the skin.

Twenty years ago, Hadgraft et al. [4] studied the mechanisms of action of Azone^®^ and some analogues. Among them, the compound identified as N-0915 (3-dodecanoyloxazolidin-2-one) showed a retarder rather than an enhancer ability. The retarding effect of N-0915 was attributed to interactions between polar head groups, specifically the two oxygen containing groups of N-0915 that enable the formation of hydrogen bonds with lipid head groups, with the possibility of interaction on both sides, then condensing the SC lipids. As AGs possess a long alkyl chain (16 and 18 carbons), it is expected they have a mode of action similar to compound N-0915, as it is able to be inserted easily into the lipid bilayer structure and allows the H-bonding of the two hydroxyl groups with adjacent polar head lipids, tightening and condensing the arrangement of SC lipids, which retards the permeation of the drugs.

In order to confirm the effect of AGs on drug transport, TEWL measurements were performed. The measurement of TEWL is a well-established method in dermatology to assess, in vivo or in vitro, the integrity of the skin barrier [17]. When the skin is impaired, an increase in water loss occurs [18,19]. In this work, skin was treated with Transcutol P**^®^**, batyl alcohol/Transcutol P**^®^**, or chimyl alcohol/Transcutol P**^®^**. As shown, treatment of the skin with Transcutol P**^®^** resulted in a TEWL increase of about 64.6%, compared with untreated skin. This increase could be attributed to the hygroscopic effect of Transcutol P**^®^** on the skin. In a previous work [20], we have demonstrated that Transcutol P**^®^** increases donor hydration by improving the outflow of water from the skin. Therefore, the well-known penetration enhancing activity of Transcutol P**^®^** has been attributed, on the one hand, to its hygroscopic properties, being capable of absorbing water and therefore changing the composition of the vehicle, which can improve the skin penetration of certain drugs. On the other hand, this penetration enhancing activity has also been attributed to its own permeation through the skin. However, treatment with batyl alcohol/Transcutol P**^®^** or chimyl alcohol/Transcutol P**^®^** decreased TEWL values in 20% and 16.6%, respectively, compared with Transcutol P**^®^**. This leads one to think that the skin became less permeable with this combination than that when treated with Transcutol**^®^** alone.

ATR-FTIR spectroscopy is a very useful technique for analyzing tissue samples without the need for further preparation by placing the sample directly on a ZnSe crystal. ATR-FTIR spectroscopy provides information about the molecular effects of formulations or vehicles on the conformational changes of SC lipid and protein domains. In this work, ATR-FTIR spectroscopy was used in order to gain a deeper insight into the structural effects caused by treating the skin with batyl and chimyl alcohols [21,22]. Of particular interest are the IR absorbances originating from the CH_2_ symmetric and asymmetric stretching vibrations of the SC lipid alkyl chains at about 2850 cm^−1^ and 2920 cm^−1^, respectively. The position and/or the bandwidth of the peaks related to these stretching vibrations are sensitive markers of both lipid conformational order and acyl chain packing [10,23]. Figure 6A shows the characteristic spectrum of untreated SC, as well as the spectra after treatment with Transcutol P**^®^**, batyl alcohol/Transcutol P**^®^**, and chimyl alcohol/Transcutol P**^®^**. As seen (Figure 6B), no changes were observed in the symmetric stretching vibration, but a shift towards lower wavenumbers was found in the asymmetric band with all formulations—markedly so with batyl and chimyl alcohols. A phase transition from an ordered state to one disordered, i.e., a liquid crystalline phase, is characterized by an increase in the rotational *gauche* isomers along the alkyl chains, which appears as a shift of asymmetric and symmetric stretching vibrations towards greater wavenumbers in the IR spectrum [23,24]; however, if a shift towards lower wavenumbers is observed, this reflects a more ordered structure and consequently a more efficient barrier [23,25]. In this sense, it can be assumed that the insertion of AGs between intercellular lipids causes a higher ordered lipid structure, likely reducing the *gauche* isomers ratio and increasing the *trans* isomers. These results confirm the retarding effect observed in the permeations assays.

## 4. Materials and Methods

### 4.1. Materials

Diclofenac, piroxicam, and naproxen were provided by Global Chemical (Bangpoo Samut Prakarn, Thailand). Glycyrrhizic acid ammonium salt and batyl alcohol were purchased from Sigma-Aldrich (St. Louis, MO, USA). Chimyl alcohol was obtained from Pfaltz & Baver (Waterbury, CT, USA). Transcutol P**^®^** was a donation from Gattefossé (Lyon, France). Sodium hydroxide, monobasic potassium phosphate, and methanol were all supplied by Fermont (Mexico). Distilled water was obtained from a RiOs™ distiller (EMD Millipore, Billerica, MA, USA).

### 4.2. Methods

In the present work, three analgesic drugs diclofenac (DI), naproxen (NP), and piroxicam (PI), were selected as model drugs to carry out the permeation tests. Additionally, a triterpenic saponin, glycyrrhizic acid ammonium salt (GLYAC), well known for its antiviral properties, was also used. Two alkylglycerol (AGs) derivatives, batyl alcohol and chimyl alcohol, were selected as modulating agents. Transcutol P**^®^** was selected as the vehicle in order to have both AGs and the drug in solution administered together, without performing a pre-treatment.

#### 4.2.1. In Vitro Skin Permeation Studies

Skin samples from a pig’s ear were used in the permeation studies. The ears were obtained from a slaughterhouse. First, skin membranes approximately 700–750 µm in thickness were prepared by dermatoming on the dorsal surface of the skin that was previously removed from the pig’s ear. Then, each skin membrane was mounted in a Franz-type glass diffusion cell (diffusion area 0.75 cm^2^) with the SC side facing the donor chamber. The receptor chamber was filled with 1.5 mL of phosphate buffer solution (PBS, pH 7.4), and it was immersed in a thermostated bath at 37 ± 0.1 °C. It allowed the temperature of the skin surface to be maintained at 32 °C (the temperature was measured using a non-contact infrared thermometer, ST350). The receptor solution was kept under continuous magnetic stirring at 500 rpm. The donor chamber of the cells was filled with 500 µL of the following solutions: (a) drug (1% *w*/*v*) in Transcutol P^®^; (b) drug (1% *w*/*v*) + batyl alcohol (1% *w*/*v*) in Transcutol P^®^; and (c) drug (1% *w*/*v*) + chimyl alcohol (1% *w*/*v*) in Transcutol P**^®^** (Transcutol**^®^** allowed dissolution of both drugs and AGs). Donors were covered to minimize the evaporation of the solvent. Permeation was carried out by sampling the receptor solution at predetermined time intervals over 24 h. At the end of the experiment and after the skin was cleaned with a cotton cloth soaked in methanol, the skin was finely divided and immersed for 24 h in 5 mL of a suitable solvent (methanol, in the case of piroxicam, naproxen, and diclofenac; PBS pH 7.4, for glycyrrhizic acid) under constant stirring at room temperature to extract the drug. Solutions were filtered through a 0.45 μm Millipore membrane. Concentration of each drug was determined by spectrophotometry (U-500 Hitachi, Hefei, AH, China) using a previously validated method. Experiments were performed in six replicates.

#### 4.2.2. Transepidermal Water Loss (TEWL) Measurement

Skin samples were prepared and mounted on modified Franz cells as described in the previous section. The receptor chamber was filled with PBS (pH 7.4). Then, 500 µL of a 1% (*w*/*v*) solution of each AGs (batyl or chimyl), using Transcutol P**^®^** as solvent, were placed in the donor chamber. After 3 h of contact, the AGs solution was completely removed from the donor chamber, gently wiping the skin with a cotton cloth soaked in methanol. A delay of an hour was left before measuring the water loss using a Tewameter (Courage + Khazaka TM210, Köln, Germany). Two controls were also tested: (1) a skin sample treated in a similar manner with 500 µL of pure Transcutol P**^®^**; and (2) a skin sample without any treatment. TEWL measurements were carried out within an acrylic cabinet, where humidity and temperature were constantly recorded [26]. Measurements were performed at 25 °C and 40% RH. Experiments were conducted in triplicate.

#### 4.2.3. Attenuated Total Reflectance-Fourier Transform Infrared (ATR-FTIR) Spectroscopy

The skin was treated with the two AG derivatives, following the procedure described in Section 4.2.2, in order to evaluate the ability of AGs to interact with lipids of the SC. Once removed from the Franz cell, the skin was placed on a horizontal ATR zinc selenide cell to record the infrared spectrum of the SC using infrared equipment (ABB-MB3000 FTIR, Quebec, QC, Canada). Measurements represented an average of 16 scans with a resolution of 8. To determine the effect of AGs, the frequencies of the peaks assigned to the C–H_2_ symmetric and asymmetric stretching vibrations of the SC lipid alkyl chains (approx. 2850 cm^−1^ and 2920 cm^−1^, respectively) were examined in the acquired spectra. Experiments were performed in triplicate.

#### 4.2.4. Statistics

The results are expressed as the mean ± standard deviation. A Student’s *t* test or an analysis of variance was performed through Statgraphics in order to test the level of significance. A multiple range test was used to locate the source of the difference when a significant *F* value was found.

## 5. Conclusions

Absorption modulators offer a potential value in drug delivery for topical and transdermal systems. However, while penetration enhancers have been studied extensively, little information is available regarding penetration retarders and their mechanisms of action.

The effect of AGs on the barrier properties of the skin is reported for the first time in this work. The results obtained with TEWL and ATR-FTIR spectroscopy are consistent with those of permeation experiments, suggesting that the presence of AGs renders a more efficient barrier, which translates to a reduced drug permeation. This may have important implications for those formulations whose aim is to restrict the location of drugs in the outermost layers of the skin, reducing transdermal penetration in order to achieve a specific local effect on the skin. However, it is important to keep in mind that the effect of a modulator is strongly dependent on the solvent used and on the physicochemical properties of the permeant. Therefore, besides TEWL, ATR-FTIR, and permeation studies, other techniques should be used to further elucidate the mechanism of action of these agents, evaluating its effect on other drug molecules and trying other solvents different to Transcutol P^®^.

## Figures and Tables

**Table 1:** Physicochemical properties of the model drugs tested in this work.

Name	Molecular Weight	p*K*_a_	Log P	Water Solubility
Diclofenac	296.20	4.15	4.51	2.37 mg/L
Naproxen	230.20	4.15	3.18	15.9 mg/L
Piroxicam	331.34	6.30	3.06	23 mg/L
Glycyrrhizic acid ammonium salt	822.94	3.98, 4.62, 5.17 ^1^	2.80	Freely sol in hot water

Diclofenac (PubChem CID: 3033); Naproxen (PubChem CID: 156391); Piroxicam (PubChem CID: 54676228); glycyrrhizic acid (PubChem CID: 14982) from https://pubchem.ncbi.nlm.nih.gov/ (NCBI PubChem Compound Database). ^1^ Taken from [10].

## Abbreviations

The following abbreviations are used in this manuscript:

AGs	alkylglycerols
ATR-FTIR	attenuated total reflectance-Fourier transform infrared
DI	diclofenac
ER	enhancement ratio
GLYAC	glycyrrhizic acid ammomium salt
NP	naproxen
PI	piroxicam
SC	stratum corneum
TEWL	transepidermal water loss
